# A Simple Physical Model Predicts Small Exon Length Variations

**DOI:** 10.1371/journal.pgen.0020045

**Published:** 2006-04-28

**Authors:** Tzu-Ming Chern, Erik van Nimwegen, Chikatoshi Kai, Jun Kawai, Piero Carninci, Yoshihide Hayashizaki, Mihaela Zavolan

**Affiliations:** 1 Division of Bioinformatics, Biozentrum, University of Basel, Basel, Switzerland; 2 Genome Exploration Research Group (Genome Network Project Core Group) RIKEN Genomic Sciences Center, RIKEN Yokohama Institute, Yokohama, Japan; 3 Genome Science Laboratory, Discovery Research Institute, RIKEN Wako Institute, Wako, Japan; The Jackson Laboratory, US; MRC-Harwell, UK; NHGRI-NIH, US; Lawrence Livermore National Laboratory, US; The Jackson Laboratory, US

## Abstract

One of the most common splice variations are small exon length variations caused by the use of alternative donor or acceptor splice sites that are in very close proximity on the pre-mRNA. Among these, three-nucleotide variations at so-called NAGNAG tandem acceptor sites have recently attracted considerable attention, and it has been suggested that these variations are regulated and serve to fine-tune protein forms by the addition or removal of a single amino acid. In this paper we first show that in-frame exon length variations are generally overrepresented and that this overrepresentation can be quantitatively explained by the effect of nonsense-mediated decay. Our analysis allows us to estimate that about 50% of frame-shifted coding transcripts are targeted by nonsense-mediated decay. Second, we show that a simple physical model that assumes that the splicing machinery stochastically binds to nearby splice sites in proportion to the affinities of the sites correctly predicts the relative abundances of different small length variations at both boundaries. Finally, using the same simple physical model, we show that for NAGNAG sites, the difference in affinities of the neighboring sites for the splicing machinery accurately predicts whether splicing will occur only at the first site, splicing will occur only at the second site, or three-nucleotide splice variants are likely to occur. Our analysis thus suggests that small exon length variations are the result of stochastic binding of the spliceosome at neighboring splice sites. Small exon length variations occur when there are nearby alternative splice sites that have similar affinity for the splicing machinery.

## Synopsis

It has recently become clear that splice variation affects most mammalian genes. It is, however, less clear to what extent these splice variations are functional and regulated by the cell as opposed to simply a result of noise in the splicing process.

One of the most frequently observed forms of splice variation are small variations in exon length in which the boundary of an exon is shifted by small amounts between different transcripts. In this work the authors study the statistics of these splice variations in detail, and the results suggest that these variations are mostly the result of noise in the splicing process. In particular, they propose a simple physical model in which the last step of splicing involves the sequence-specific binding of the splicing machinery to the splice site. In this model, small length variations can occur when there are nearby splice sites with comparable affinity for the splicing machinery. The authors show that this model not only accurately predicts the relative abundances of different splice variations but also predicts which splice sites are likely to undergo small exon length variations.

## Introduction

Anticipating that the sequencing and initial annotation of the human [1,2] and mouse [3] genomes will not be able to uncover all the complexities of mammalian gene structures, several groups have focused on producing high-quality, annotated transcript data such as the Riken Clone Collection [4,5], the Mammalian Gene Collection [6], the NCBI Reference Sequence [7], and the NCBI unfinished high-throughput cDNA sequences. In conjunction with genome sequences, these data have revealed that alternative splicing [1,8–11], alternative transcriptional initiation, and alternative polyadenylation [5,12,13] are extremely common, affecting over 70% of mammalian genes [14].

Many factors at the molecular level appear to play a role in the regulation of splicing, from the recognition of the primary splice signals by the components of the spliceosome, to modulation of splicing via interactions of signaling and RNA processing pathways with the spliceosome. More specifically, the choice of splice site appears to be determined by a combination of (1) the strength of the splice signal, i.e., the affinity for the splicing machinery of the sequence around the splice site, (2) structural constraints set on the interactions of spliceosomal components by the lengths and sequences of introns and exons and possibly by the secondary structure of the mRNA, (3) the presence of enhancer or repressor elements that may serve, respectively, to activate a weak splice site or repress a strong one, and (4) the effective concentrations of splicing factors such as SR proteins and heterogeneous nuclear ribonucleoproteins that can be regulated through post-translational modifications such as phosphorylation [15]. The extensive literature elaborating on the mechanisms of repression and activation of specific splice sites has been reviewed by Smith and Valcarcel [16] and more recently by Matlin et al. [17].

The high estimates of the frequency of alternative splicing in human [8,14,18] and mouse [13] genes raise the question to what extent these splice variations are functional, with their production controlled and regulated by the cell, versus being the result of inherent noise in the molecular process of splicing. The molecular mechanisms mentioned in the previous paragraph are all susceptible to noise, e.g., thermodynamic noise, fluctuations in the concentrations of splicing factors, fluctuations in elongation rates, and any other fluctuations that are not under the control of the cell. It is thus clear that some of the splice variation observed in the sequence data might simply be a result of noise [19]. At the same time, one can easily imagine that many of the molecular mechanisms just mentioned could be exploited by the cell to regulate the expression of different splice variants under different conditions.

In a previous study [13], we found that the second most common form of splice variation (after “cassette” or “alternative” exons that are included in some but not all transcripts) is a small change in exon length due to the use of closely spaced alternative donor or acceptor sites. Intuitively, the simplest explanation for these abundant small exon length variations is that they are a result of noise in the splicing process that causes the spliceosome to “slip” by a small number of nucleotides, perhaps to a competing neighboring splice site. However, as we previously reported [13], three-nucleotide variations at tandem acceptor sites are by far the most common among these small exon length variations, and are much more common than any other exon length variation. This seems to suggest that processes other than simple noise must be causing these small in-frame shifts. Indeed, in this context Hiller et al. [20] have proposed an intriguing hypothesis, namely, that splice variations involving only three nucleotides at so-called NAGNAG tandem acceptor sites are introduced in a regulated manner to “fine-tune” the protein sequence. More recently, these three-nucleotide splice variations at NAGNAG sites have attracted considerable attention [21], including two papers [22,23] that appeared after our submission of the current work.

Here we extensively study the statistics of small exon length variations. We revisit our original hypothesis that these small exon length variations are a result of noise in the splicing process. In particular, we show that a combination of the effects of nonsense-mediated decay (NMD) and a simple physical model of the splicing machinery binding in a stochastic manner to nearby splice sites can efficiently explain all the observed statistics. In addition, we show that our physical model can predict which NAGNAG tandem acceptor sites are likely to undergo alternative splicing, which will splice exclusively at the first NAG, and which will splice only at the second NAG.

## Results

### Splice Variations at Acceptor and Donor Sites

**Figure 1:**
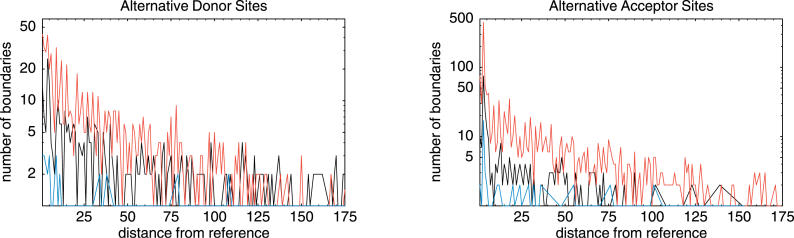
The Number of Splice Events Involving Alternative Donor and Acceptor Sites at a Specified Distance Relative to the Reference (Most Commonly Used) Splice Site The horizontal axis shows the distance from the reference splice site corresponding to each genomic exon for both donor sites (left) and acceptor sites (right). The red lines correspond to coding exons, the black lines to UTR exons, and the blue lines to exons from non-protein-coding transcription units. The vertical axis is shown on a logarithmic scale.

The use of alternative splice donor and acceptor sites leads to exons whose length varies between transcripts. By far the most common variation of this kind is a difference of precisely three nucleotides at acceptor sites. To investigate the origin of such variations we selected all exons that showed variation at only one of their two splice sites, i.e., only at their acceptor site or only at their donor site. For each exon with an alternative acceptor (or donor) site we chose the most common splice site as a reference site and counted the total number of alternative splice events at different distances from the reference site. Figure 1 shows the distribution of distances for both acceptor and donor sites, calculated separately for coding, untranslated region (UTR), and noncoding exons.

The first thing to note is that the total number of alternative acceptor sites is larger than the overall number of alternative donor sites. This observation is consistent with our previous reports on the FANTOM2 dataset [13] as well as with the observation made by Sugnet et al. [24] that conserved mouse and human alternative acceptor splice sites are twice as common as conserved alternative donor splice sites.

The second thing to note is that small length variations are very common: 23.7% of all donor site variations and 43.7% of all acceptor site variations involve ten or fewer nucleotides. This is suggestive of a “noise” process in which the spliceosome “slides” a few nucleotides from its initially chosen position.

### Preference for Reading Frame Preservation and NMD

**Figure 2:**
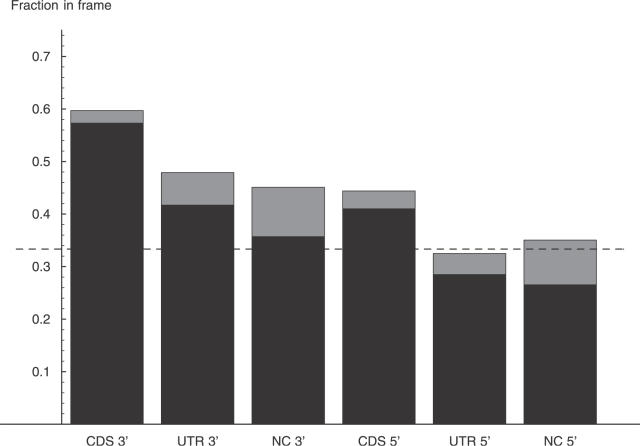
Proportion of In-Frame Variations at Donor and Acceptor Splice Sites That Are Located within CDS, UTR, and Noncoding Regions This figure shows the fractions of alternative splice events that lead to an in-frame shift with respect to the reference boundary at acceptor (3′) and donor (5′) splice sites of CDS, UTR, and noncoding (NC) exons. The estimated fraction is in the middle of the gray bar, with the gray bar indicating two standard errors. The dashed line shows the fraction 1/3 that would be expected by chance.

Most of the exon length variations shown in Figure 1 are not a multiple of three in length, and would therefore have dramatic effects on translation whenever the splice boundary overlaps the coding region (CDS). We will refer to exon length variations as “in-frame” and “frame-shifting” depending on whether the change in exon length is or is not a multiple of three. Figure 2 shows the fraction of in-frame and frame-shifting variations for each category of splice sites. We see that in-frame variations are overrepresented at the acceptor boundaries of all exon types. In contrast, in-frame variations are overrepresented at donor sites only of CDS exons, and they amount to roughly 1/3 of the variations at the donor sites of UTR and noncoding exons.

**Figure 3:**
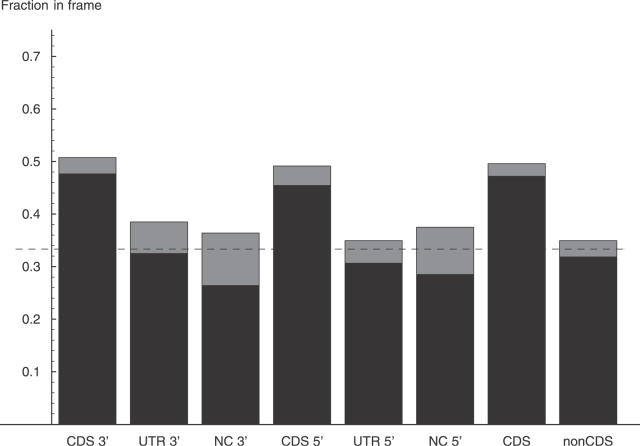
Proportion of In-Frame Variations of More Than Four Nucleotides at Donor and Acceptor Sites Located within CDS, UTR, and Noncoding Regions This figure shows the fractions of alternative splice events that lead to an in-frame shift with respect to the reference boundary at acceptor (3′) and donor (5′) splice sites of CDS, UTR, and noncoding (NC) exons, when only splice events that are more than four nucleotides shifted with respect to the reference boundary are considered. The two rightmost columns show the fractions when the data from all CDS exons and all non-CDS exons are pooled. The estimated fraction is in the middle of the gray bar, with the gray bar indicating two standard errors. The dashed line shows the fraction 1/3 that would be expected by chance.

The different behavior of donor and acceptor sites is the result of the very different distribution of very small exon length variations of 1–4 nucleotides. As we show in detail below, the frequencies of these very small exon length variations at acceptor and donor sites are the result of the different sequence composition of the first few intronic bases at donor and acceptor sites. If we focus on exon length variations of more than four nucleotides, we find that, strikingly, both donor and acceptor splice sites show the same pattern of in-frame variation across exon types (Figure 3). Namely, the frequency of in-frame variations at both donor and acceptor splice sites of noncoding and UTR exons is statistically indistinguishable from 1/3, which is what one would expect by chance. Moreover, CDS exons show the same overrepresentation (approximately 48%) of in-frame variations at both donor and acceptor splice sites.

**Figure 4:**
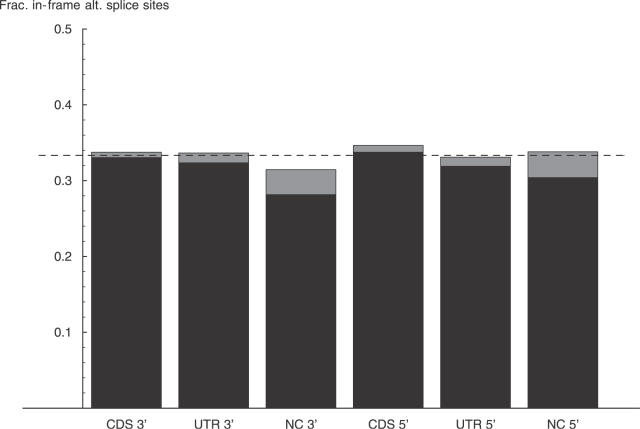
Proportion of Putative Donor (GT) and Acceptor (AG) Splice Sites That Are Located In-Frame Relative to the Splice Sites in CDS, UTR, and Noncoding Regions This figure shows the fraction of AG dinucleotides that occur at distance that is a multiple of three in the first 100 intronic bases upstream of acceptor (3′) splice sites of exons that show splice variation at their acceptor sites, and the fraction of GT dinucleotides that occur at a distance that is a multiple of three in the first 100 intronic bases downstream of donor (5′) splice sites of exons that show splice variations at their donor sites. Occurrences of AG or GT within the first four bases flanking the splice sites were not counted. The estimated fraction is in the middle of the gray bar, with the gray bar indicating two standard errors. The dashed line shows the fraction 1/3 that would be expected by chance.

One possible explanation for the overrepresentation of in-frame variations could be that the sequences flanking CDS exon boundaries are biased such that alternative splice sites occur more often in frame than out of frame. To test this hypothesis we extracted the 100 nucleotides of the intronic sequence flanking the acceptor splice site of each exon that shows length variation at the acceptor boundary and counted the number of times an AG dinucleotide occurs at different distances from the boundary. Similarly, we counted the number of times the dinucleotide GT occurs at different distances from donor sites of exons that show variation at their donor site. We then determined the fraction of times AG and GT occur in frame relative to the acceptor and donor splice sites, respectively. The results are shown in Figure 4. We see that, for both donor and acceptor sites, and for all exon types, the frequency of in-frame occurrence of dinucleotides that could form alternative splice sites is very close to 1/3. It thus appears that biases in the sequence composition flanking CDS exons cannot explain the overrepresentation of in-frame exon length variations at either donor or acceptor sites.

The most plausible explanation for the statistics of the in-frame variations is that NMD removes a fraction of transcripts that have frame-shifting exon length variations in CDS exons. The details of the NMD process are not completely understood, but it is generally thought to function as follows [25,26]. After the splicing process, the exon junction complexes remain attached and are carried along with the transcript. During a preliminary round of translation these complexes are removed by the translation machinery. If any such complex remains further than some critical distance away from the stop codon, the transcript is targeted by NMD. Thus, frame-shifting length variation in CDS exons leads to premature stop codons, which in turn increase the chance of the transcript being targeted by NMD. In contrast, the boundaries of noncoding and UTR exons already do not overlap the CDS; therefore, the shifts that occur here do not alter the probability of the transcript being targeted by NMD.

In summary, it is reasonable to assume that some fraction of all frame-shifting exon length variations at CDS exons are targeted by NMD, and that only a fraction *f* survive NMD. Let us assume that before NMD a fraction ρ_i_ of all exon length variations at CDS exons are in frame. Since in-frame exon length variations are not affected by NMD, and a fraction *f* of frame-shifting variations make it past NMD, it follows that the observed frequency ρ_o_ of in-frame exon length variations in CDS exons is given by


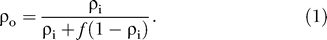


There is no reason to believe that the fraction ρ_i_ of in-frame variations before NMD is different from 1/3. This assumption is supported by the fact that the fraction of potential acceptor and donor dinucleotides within 100 nucleotides of exon boundaries is 1/3 for both CDS and non-CDS exons (see Figure 4). We thus assume that ρ_i_ = 1/3 for CDS exons as well. Since ρ_o_ = 0.484 (Figure 3) for CDS exons, it then follows that *f* = 0.53. That is, the data suggest that slightly more than 50% of the transcripts that contain a frame-shifting exon length variation in a CDS exon survive the NMD process.

### Exon Length Variations of 1–4 Nucleotides and NAGNAG Acceptor Boundaries

**Figure 5:**
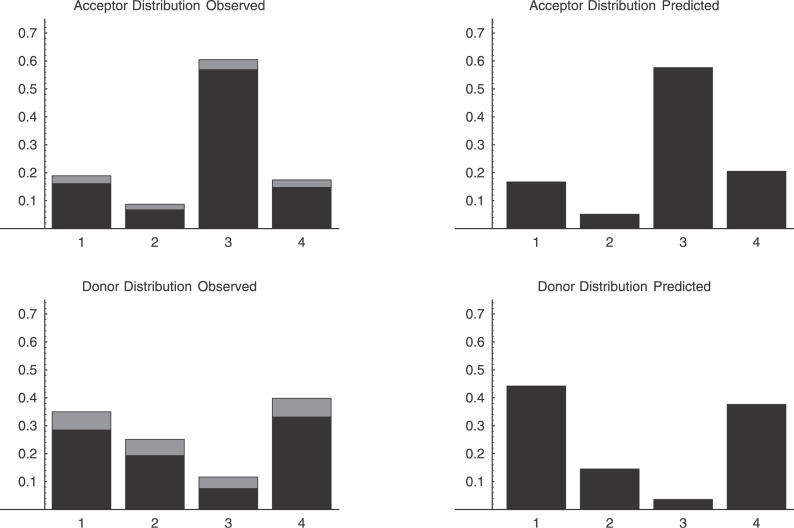
The Distribution of Alternative Splice Events That Are Shifted by One, Two, Three, or Four Nucleotides with Respect to the Reference Splice Site The two left panels show the observed distributions at acceptor sites (above) and donor sites (below). The estimated relative frequency is in the middle of the gray bar, with the width of the gray bar corresponding to two standard errors. The panels on the right show the predicted relative frequency of alternative splice events of lengths 1–4 based on the splice site WMs and the sequences around exon boundaries that show splice variation.

We now turn to the exon length variations of 1–4 nucleotides, whose relative frequencies are shown in Figure 5. To take into account the effects of NMD we have rescaled the numbers of variations of length three by a factor *f* = 0.53, as calculated in the previous section. Moreover, since the frequencies of such variations at CDS, UTR, and noncoding exons are very similar, we have pooled the data for each type of splice site (Table S1 lists these distributions separately for each exon type). Figure 5 thus shows estimates of the relative frequencies of splice variations of lengths 1–4 nucleotides before NMD, which appear to be very different for donor compared to acceptor splice sites. At donor sites, the most common variations are shifts of one or four nucleotides while in-frame variations of length three are rare. In contrast, at acceptor sites the in-frame variations of length three are highly overrepresented, as we have already reported in our analysis of the FANTOM2 dataset [13]. The very large majority (94%) of these three-nucleotide variations involve tandem acceptor sites whose sequence is of the form NAGNAG. These have been the topic of a recent paper by Hiller et al. [20], who proposed that the role of such variations is to “fine-tune” protein forms by addition/deletion of a single amino acid. That is, Hiller et al. [20] suggested that these NAGNAG sites have been specifically selected to provide the cell with alternative protein forms, and to express these different protein forms in a regulated manner.

If the three-nucleotide variations at NAGNAG acceptor sites were indeed important for fine-tuning protein forms, then one would expect these variations to be more abundant at CDS than at non-CDS exons. However, once we correct for the overrepresentation of *all* in-frame variations due to NMD, this is not what we observe. As shown in Table S1, the relative frequency of three-nucleotide variations is not significantly different at CDS, UTR, and noncoding exons. In addition, the NAGNAG sequence motif is not overrepresented at acceptor sites of CDS exons. On the contrary, the frequency of NAGNAG sites at the splice boundaries of UTR and noncoding exons is 6.7% compared to only 5.9% at the boundaries of coding exons. This is statistically significantly lower in a χ^2^ test at a *p*-value of 0.00014. Thus, NAGNAG sequences are in fact a little less frequent at the acceptor sites of CDS exons than at those of non-CDS exons.

We next investigated the evolutionary conservation of NAGNAG acceptor sites. If the NAGNAG sites that show splice variation were explicitly selected to do so, one would expect them to be better conserved evolutionarily than NAGNAG sites in exons that show no splice variation. To test this hypothesis, we extracted the human sequences that correspond to NAGNAG sites in mouse from the pairwise mm5–hg17 alignments provided by the University of California Santa Cruz Genome Bioinformatics group (http://hgdownload.cse.ucsc.edu/goldenPath/mm5/vsHg17/axtNet).

We found that the proportion of mouse NAGNAG acceptor sites that have corresponding human NAGNAG sites (allowing the nucleotides at the N positions to vary) is in fact slightly higher in invariant exons (59.5%) than in variant exons (54.7%) (3,998/6,715 versus 273/499, χ^2^ test, *p* = 0.04). The frequency of perfectly conserved NAGNAG sites (where the nucleotides at the N positions are conserved as well) is 45.7% in invariant exons and 42.7% in variant exons, which statistically is not significantly different. Thus, NAGNAG sites of variant exons are not more conserved in evolution than NAGNAG sites of invariant exons. If anything they are less conserved than NAGNAG sites of invariant exons. We thus cannot find any evidence that the abundance of three-nucleotide variations at acceptor sites is due to specific selection, or that it is related to the coding potential of the transcript.

We instead consider the much simpler hypothesis that the small exon length variations are a result of inherent noise in the splicing process. We hypothesize that the final step in the process of splice site selection involves the sequence-specific binding of the splicing machinery to the mRNA. This binding process is subject to thermal noise just like any physicochemical process. Whenever multiple “binding sites” with comparable affinity occur near each other, the splicing machinery may bind to these alternative sites in a stochastic fashion, and this will lead to small shifts of the splice site.

**Figure 6:**
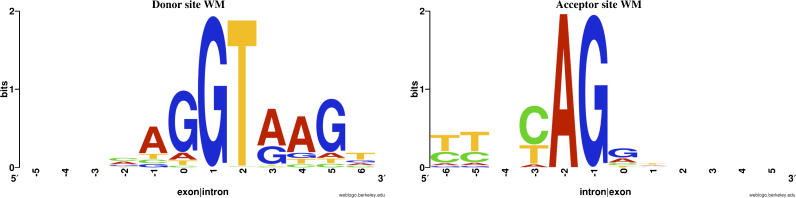
WMs Representing the Sequence Specificity of the Spliceosome at Invariant Donor and Acceptor Splice Sites WMs have been constructed from six exonic and six intronic nucleotides flanking each type of splice site. The relative sizes of the letters are proportional to the frequency *w_α_^i^* of each nucleotide α at position i. The total height in each column is given by the information score I = Σ_α_ *w_α_^i^* log(4*w_α_^i^*).

To test this hypothesis we first constructed computational models of the sequence specificity of the splicing machinery at acceptor and donor sites. Specifically, we gathered acceptor and donor boundaries of *invariant* exons and reconstructed weight matrices (WMs) of length 12 from the six intronic bases and the six exonic bases flanking each boundary. These WMs are shown in Figure 6. The WMs demonstrate the well conserved GT dinucleotide immediately following the donor boundary and the AG dinucleotide immediately preceding the acceptor boundary. The WMs also show the known preference for a second GT dinucleotide four nucleotides downstream of the donor site, the polypyrimidine tract 5–6 nucleotides upstream of the acceptor site, and the preference for a cytosine immediately preceding the AG of the acceptor site.

We now assume that the probability of the splicing machinery binding to a particular sequence is proportional to the probability of observing that sequence when sampling from the WM representing that boundary. That is, we assume that the probability *P*(*i*) that the splicing machinery will bind and splice at a particular location *i* is proportional to the probability of the local sequence *s_i_*_−6_ *s_i_*_−5_...*s_i_*_+4_ *s_i_*_+5_ under the WM


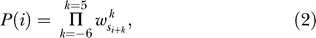


where 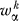 is the frequency of base α at position *k* of the WM, and *i* is the location of the putative splice site.

For the donor site WM we collected all exons that show variation at the donor site and calculated the probabilities *P*(*i*) at the positions that are shifted by between one and four nucleotides (either to the left or right) with respect to the observed splice site. By summing all *P*(*i*) that correspond to shifts of the same length, we calculated the relative frequencies of length variations of lengths 1–4 that our model predicts. These predictions are shown in the lower right panel of Figure 5. In the same way, using the acceptor site WM and the sequences flanking acceptor sites in exons that show variation at their acceptor site, we calculated the relative frequencies of variations of lengths 1–4 that our model predicts at acceptor sites. These predictions are shown in the upper right panel of Figure 5. Given the simplicity of our model, its predictions of the relative abundances of different length variations match the data surprisingly well. At the donor sites, it correctly predicts that shifts of length one and four are the most abundant and that shifts of length three are the least abundant. At the acceptor sites, the model predicts relative abundances that are quantitatively very close to the observed abundances. In particular, the predicted abundance of three-nucleotide variations is within 1% of the observed abundance. We thus see that a very simple model of splice site selection based on the sequence specificity of the splicing machinery at each splice site can correctly predict the relative abundances of small exon length variations. This further supports the hypothesis that these small exon length variations are mostly the result of inherent noise in the splicing process.

### Local Sequence Distinguishes Variant Tandem Acceptor Sites from Nonvariant Tandem Acceptor Sites

If our model for the small exon length variations is correct, it should also be possible to predict, from the sequence, which acceptor sites are most likely to be prone to three-nucleotide length variations. To this end we focused on all acceptor sites that show the sequence pattern NAGNAG at their splice site. We collected all acceptor sites with sequence NAGNAG (irrespective of which of the two NAG sequences is used as splice site) and then selected only those sites for which there are at least two transcripts in the data. We then counted, for each NAGNAG site, how many times we observed splicing at the first and how many times at the second NAG site. Based on these counts we separated the NAGNAG sites into three categories: those that splice only at the first NAG, those that splice only at the second NAG, and those that splice at both NAGs.

We then investigated to what extent we could predict the category of each NAGNAG site by using the WM constructed from the invariant acceptor sites. We again assumed that the binding affinity of the splicing machinery to a putative acceptor site sequence is proportional to the log-likelihood of the putative acceptor site sequence given the acceptor site WM. If we additionally assumed that the probability of splicing occurring at the different NAG sites is proportional to the equilibrium frequencies with which the splicing machinery binds at these sites, then the category of a NAGNAG site is only a function of the difference in log-likelihood of the neighboring acceptor sites.

**Figure 7:**
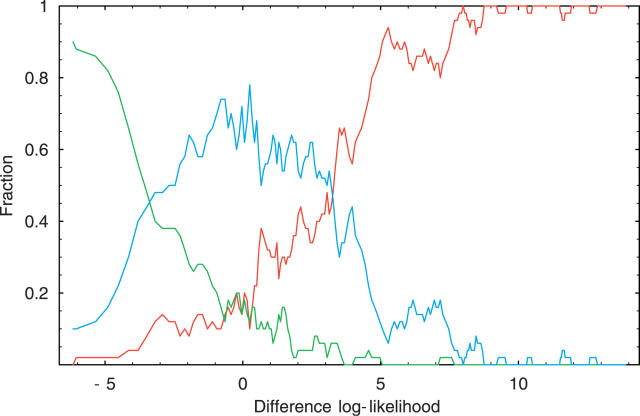
Dependency of the Frequency of Alternative Splicing at NAGNAG Sites on the Relative Likelihood of the Two Putative Acceptor Sites The figure shows the fraction of all NAGNAG boundaries that splice only at the first NAG (red), only at the second NAG (green), or at both NAGs (blue) as a function of the log-likelihood difference of the first and second putative splice sites for the acceptor site WM.

Figure 7 shows the fractions of NAGNAG sites that splice only at the first NAG, only at the second NAG, or at both NAGs as a function of the log-likelihood difference of the two sites. The results are quite striking. We see that, using the simple WM model, one can reasonably accurately predict which NAGNAG sites splice only at the first NAG, which splice only at the second, and which show three-nucleotide length variations. Whenever the log-likelihood of the first site is larger by five or more than the log-likelihood of the second site, then splicing virtually always occurs at the first site only. Similarly, if the log-likelihood of the second site is larger by five or more than the log-likelihood of the first site, then splicing virtually always occurs at the second site. When there is almost no difference in the log-likelihood of the two sites, then in the large majority of examples one observes splicing at both sites. These results further support our hypothesis that splice site selection is simply based on the affinity of the splice site for the splicing machinery, and that small exon length variations occur whenever there are neighboring splice sites with comparable affinity.

## Discussion

Recent large-scale sequencing efforts have made clear that the great majority of mammalian genes are subject to splice variation, and that some genes show a very large number of different transcript forms. One of the most basic questions to ask about these splice variations is to what extent they are regulated by the cell rather than being the result of noise in the molecular process or of other fluctuations that are not controlled by the cell. We investigated this issue for small exon length variations caused by the alternative usage of nearby acceptor and donor sites.

Small exon length variations are the second most common form of splice variation. Among these, three-nucleotide variations at tandem acceptor sites containing a NAGNAG sequence pattern are by far the most common. It has been suggested that cells can “fine-tune” the expression of protein forms through these subtle splice variations [20]. However, we were unable to find any evidence that NAGNAG sites are either under specific evolutionary selection or are more abundant at coding exons than at noncoding exons. In contrast, we collected and compared a number of statistics on observed small exon length variations, and showed that all these statistics can be accurately explained by the combined effect of NMD and the stochastic binding of the splicing machinery to competing nearby splice sites.

Our analysis of the relative abundances of exon length variations of five and more nucleotides at donor and acceptor sites of CDS, UTR, and noncoding exons strongly suggests that NMD targets about 50% of frame-shifting exon length variations at CDS exons. This effect partly explains the overrepresentation of three-nucleotide variations in the data. When the effects of NMD are taken into account, donor and acceptor sites show very distinct relative abundances of small exon length variations, with an overrepresentation of shifts of length one and four at donor sites, and an overrepresentation of shifts of length three at acceptor sites. However, shifts of length three at acceptor sites are not more abundant at CDS exons than they are at UTR and noncoding exons. This observation again supports our conjecture that the abundance of three-nucleotide shifts is special to the specifics of splicing at acceptor sites and is not related to the coding potential of the exon.

To explain the relative abundances of these small exon length variations we introduced a model that assumes that the last step in splice site selection involves the sequence-specific binding of the splicing machinery to a splice site and that the probability of splicing occurring at a particular site is proportional to the affinity of the splice site sequence for the splicing machinery. In this model, small exon length variations can occur when there are multiple binding sites with comparable affinity near each other. Our model is similar to the scanning model proposed by Smith et al. [27] for the selection of 3′ splice sites: the spliceosome recognizes and binds the region of the branch point and polypyrimidine sequence, but once there, it can still “see” a limited stretch of sequence in which competing splice sites may be present. The relative frequencies with which these competing splice sites are used in the splicing reaction are determined by the affinity of the spliceosome for the sequences of the different sites.

From the splice sites of invariant exons we constructed WMs representing the sequence specificity of the splicing machinery at donor and acceptor sites. We used these WMs to predict the relative frequencies of small exon length variations from the sequences flanking the observed splice sites of variant exons and found that the predictions accurately reproduce the observed relative abundances. For example, the relatively high abundance of shifts of four nucleotides at donor sites is explained by the common occurrence of a GT pattern at position +5 of the intron [13,28]. The relatively high abundance of three-nucleotide variations at acceptor splice sites is explained by the fact that the WM of the acceptor sites strongly disfavors a guanine at position −3 (directly upstream of the AG) and that positions −5, −6, and further upstream are part of the polypyrimidine tract that disfavors the occurrence of purines in general. Therefore, whenever an alternative AG dinucleotide does occur, it is almost always at position −3 and not at −1, −2, or −4.

Apart from explaining the relative abundances of small exon length variations at both donor and acceptor sites, our simple model also predicts, with reasonable accuracy, which NAGNAG acceptor sites show splice variation and which do not. We showed that when one of the two neighboring sites has much higher affinity than the other, one observes splicing almost exclusively at the site with the higher affinity. When the neighboring sites have similar affinity, three-nucleotide variations are observed in the large majority of cases.

## Materials and Methods

### Transcript mapping and splice analysis.

The transcripts used in the study consist of the 102,797 FANTOM3 mouse full-length cDNAs [29] and 52,070 GenBank mouse mRNAs. We mapped all these transcripts to the mm5 assembly of the mouse genome available from the University of California Santa Cruz.

For the identification of splice variants we used a new implementation of the automated splicing analysis pipeline that we developed for the FANTOM2 project [13]. Briefly, we first mapped all cDNAs to the mouse genome using our novel spliced alignment algorithm, SPA [30], which produces better quality alignments than other commonly used cDNA-to-genome alignment programs. In particular, it has fewer alignment errors around splice boundaries, and has a better coverage of the 5′ and 3′ ends of the cDNAs. The details of the comparisons of SPA's mappings to those of other methods are described in van Nimwegen et al. [30].

To avoid biases from transcripts that are badly mapped we selected only those transcripts that had at least 75% of their nucleotides mapped to the genome, with at least 95% identity or fewer than ten mismatches in each exon. This procedure yielded 129,655 mapped transcripts, which we clustered such that the mapping of each transcript in a cluster shared at least one exonic nucleotide on the same strand with at least one other transcript in the cluster [11,13]. We obtained 42,023 clusters (transcription units) that we analyzed for splice variation. We refer the reader to Zavolan et al. [13] for the details of the annotation procedure. Briefly, all exons whose genomic mappings overlap were clustered into “genomic exons.” For each genomic exon we then compared the set of exons corresponding to it to identify splice variation.

For our analysis of exon length variations we extracted all genomic exons that show only variation at their donor splice site and all exons that show only variation at their acceptor splice site. For each such genomic exon we then extracted the set of all “clean” exons corresponding to it. These “clean” exons were selected based on the following criteria: (1) the first and last ten nucleotides of every clean exon must be perfectly aligned (no mismatches or gaps) to the genome, and (2) the first ten nucleotides of the flanking exon(s) must be perfectly aligned. We used the FANTOM3 and GenBank annotation of CDSs to separate the exon boundaries of the clean exons into boundaries that overlap with the CDS, boundaries that are located in the UTRs of transcripts that have a CDS annotated, and boundaries of exons from noncoding transcription units. A noncoding transcription unit has no CDS annotated for any of its corresponding transcripts.

For each genomic exon with variation only at the acceptor site or only at the donor site we then determined the number of times each alternative boundary was observed and took the most abundant boundary as the “reference boundary.” We then counted the total number of times other boundaries were observed for each of these exons, and recorded the distances of these alternative boundaries from the reference boundary. Finally, we constructed from these counts the histograms of the number of observed exon length variations as a function of the distance to the reference boundary for each boundary type and each class of exon. The total numbers of observed exon length variations for donor sites were 871 in CDS exons, 524 in UTR exons, and 117 in noncoding exons, and for acceptor sites were 1,620 in CDS exons, 366 in UTR exons, and 109 in noncoding exons.

The relatively low number of variations in noncoding exons is a result of the fact that transcripts from noncoding transcription units in general have far fewer exons than do coding transcripts. In addition, there are many transcripts for which no CDS is annotated but that occur in a transcription unit that does contain at least one transcript with an annotated CDS. We exclude these transcripts because it is unclear whether they are indeed noncoding or whether their CDS has simply not been annotated.

For every exon that has variation only at its acceptor boundary we collected all transcripts that contain this exon and extracted the first 100 intronic nucleotides upstream of the acceptor site for this exon. Similarly, for every exon with variation only at its donor boundary we collected all transcripts that contain this exon and extracted the first 100 nucleotides downstream of the donor site in each of these transcripts.

### Selection of the sequences used for constructing the WMs for donor and acceptor splice sites.

We used the full set of clean invariant exons to extract sequences at the acceptor and donor splice sites. We removed all sequences that contained ambiguous characters and obtained a set of 130,827 sequences in each set. We wanted to use the sequences of these invariant exons to construct WMs for the acceptor and donor site sequences. However, since we also wanted to score NAGNAG sites for these WMs, we split the set of all invariant exons into two halves and used only the first half to construct the WMs of the six intronic and six exonic nucleotides flanking the splice site. The other half we used for extracting NAGNAG sites of invariant exons.

### Extraction of NAGNAG acceptor splice sites and log-likelihood histogram.

From the second half of acceptor site sequences of invariant exons just described we collected all boundaries that have a NAGNAG sequence. There were 2,444 cases of NAGNAG invariant exons that splice at the first NAG and 228 cases of NAGNAG invariant exons that splice at the second NAG. The set of variant exons with NAGNAG sites consisted of all clean exons that contain a NAGNAG motif at their acceptor site and whose only splice variation involves the use of alternative acceptor sites precisely at the NAGNAG boundary. We obtained 404 exons with such variant NAGNAG acceptor sites.

For each of these NAGNAG sites we calculated the difference in log-likelihood of the tandem putative acceptor sites for the acceptor site WM. We then ordered all 3,076 NAGNAG sites by the log-likelihood difference (from small to large) and calculated the average log-likelihood difference and fraction of sites variant, invariant splicing at the first NAG, and invariant splicing at the second NAG in consecutive groups of 50 sites. That is, the horizontal value of the leftmost data points of the red, green, and blue curves in Figure 7 were obtained by averaging the log-likelihood differences of the first 50 NAGNAG sites, and the vertical values were obtained by calculating the fraction of variant NAGNAG sites (blue), invariant NAGNAG sites splicing at the first boundary (red), and invariant NAGNAG sites splicing at the second boundary (green) among those first 50 NAGNAG sites. Similarly, the second leftmost set of data points was obtained by calculating the same averages and fractions over NAGNAG sites 11 through 60, the third set of points over NAGNAG sites 21 through 70, etc.

## Supporting Information

Table S1 The Relative Frequencies and Two Standard Errors of Exon Length Variations of Length 1–4 at Donor and Acceptor Sites of Different Exon Types Note that, in order to correct for NMD, the number of variations of length three has been multiplied by 0.53 and rounded to the nearest integer.
(11 KB PDF)

## Abbreviations

CDS: coding region
NMD: nonsense-mediated decay
UTR: untranslated region
WM: weight matrix

## References

[pgen-0020045-b001] Lander E, Linton L, Birren B, Nusbaum C, Zody M (2001). Initial sequencing and analysis of the human genome. Nature.

[pgen-0020045-b002] Venter J, Adams M, Myers E, Li P, Mural R (2001). The sequence of the human genome. Science.

[pgen-0020045-b003] Waterston R, Lindblad-Toh K, Birney E, Rogers J, Abril J (2002). Initial sequencing and comparative analysis of the mouse genome. Nature.

[pgen-0020045-b004] Kawai J, Shinagawa A, Shibata K, Yoshino M, Itoh M (2001). Functional annotation of a full−length mouse cDNA collection. Nature.

[pgen-0020045-b005] Suzuki Y, Taira H, Tsunoda T, Mizushima−Sugano J, Sese J (2001). Diverse transcriptional initiation revealed by fine, large-scale mapping of mRNA start sites. EMBO Rep.

[pgen-0020045-b006] Gerhard D, Wagner L, Feingold E, Shenmen C, Grouse L (2004). The status, quality, and expansion of the NIH full−length cDNA project: The Mammalian Gene Collection (MGC). Genome Res.

[pgen-0020045-b007] Pruitt K, Tatusova T, Maglott D (2005). NCBI Reference Sequence (RefSeq): A curated non-redundant sequence database of genomes, transcripts and proteins. Nucleic Acids Res.

[pgen-0020045-b008] Mironov A, Fickett J, Gelfand M (1999). Frequent alternative splicing of human genes. Genome Res.

[pgen-0020045-b009] Modrek B, Resch A, Grasso C, Lee C (2001). Genome-wide detection of alternative splicing in expressed sequences of human genes. Nucleic Acids Res.

[pgen-0020045-b010] Brett D, Pospisil H, Valcarcel J, Reich J, Bork P (2002). Alternative splicing and genome complexity. Nat Genet.

[pgen-0020045-b011] Zavolan M, van Nimwegen E, Gaasterland T (2002). Splice variation in mouse full-length cDNAs identified by mapping to the mouse genome. Genome Res.

[pgen-0020045-b012] Beaudoing E, Gautheret D (2001). Identification of alternate polyadenylation sites and analysis of their tissue distribution using EST data. Genome Res.

[pgen-0020045-b013] Zavolan M, Kondo S, Schonbach C, Adachi J, Hume D (2003). Impact of alternative initiation, splicing, and termination on the diversity of the mRNA transcripts encoded by the mouse transcriptome. Genome Res.

[pgen-0020045-b014] Johnson J, Castle J, Garrett-Engele P, Kan Z, Loerch P (2003). Genome-wide survey of human alternative pre-mRNA splicing with exon junction microarrays. Science.

[pgen-0020045-b015] Stoilov P, Daoud R, Nayler O, Stamm S (2004). Human tra2-beta1 autoregulates its protein concentration by influencing alternative splicing of its pre-mRNA. Hum Mol Genet.

[pgen-0020045-b016] Smith C, Valcarcel J (2000). Alternative pre-mRNA splicing: The logic of combinatorial control. Trends Biochem Sci.

[pgen-0020045-b017] Matlin A, Clark F, Smith C (2005). Understanding alternative splicing: Towards a cellular code. Nat Rev Mol Cell Biol.

[pgen-0020045-b018] Croft L, Schandorff S, Clark F, Burrage K, Arctander P (2000). ISIS, the intron information system, reveals the high frequency of alternative splicing in the human genome. Nat Genet.

[pgen-0020045-b019] Kan Z, States D, Gish W (2002). Selecting for functional alternative splices. Genome Res.

[pgen-0020045-b020] Hiller M, Huse K, Szafranski K, Jahn N, Hampe J (2004). Widespread occurrence of alternative splicing at NAGNAG acceptors contributes to proteome plasticity. Nat Genet.

[pgen-0020045-b021] Tadokoro K, Yamazaki-Inoue M, Tachibana M, Fujishiro M, Nagao K (2005). Frequent occurrence of protein isoforms with or without a single amino acid residue by subtle alternative splicing: The case of Gln in DRPLA affects subcellular localization of the products. J Hum Genet.

[pgen-0020045-b022] Hiller M, Huse K, Szafranski K, Jahn N, Hampe J (2006). Single-nucleotide polymorphisms in NAGNAG acceptors are highly predictive for variations of alternative splicing. Am J Hum Genet.

[pgen-0020045-b023] Martin A, Yael M (2006). Alternative splicing regulation at tandem 3′ splice sites. Nucleic Acids Res.

[pgen-0020045-b024] Sugnet C, Kent W, Ares M, Haussler D (2004). Transcriptome and genome conservation of alternative splicing events in humans and mice. Pac Symp Biocomput.

[pgen-0020045-b025] Le Hir H, Gatfield D, Izaurralde E, Moore M (2001). The exon-exon junction complex provides a binding platform for factors involved in mRNA export and nonsense-mediated mRNA decay. EMBO J.

[pgen-0020045-b026] Maquat L (2004). Nonsense-mediated mRNA decay: Splicing, translation and mRNP dynamics. Nat Rev Mol Cell Biol.

[pgen-0020045-b027] Smith C, Chu T, Nadal-Ginard B (1993). Scanning and competition between AGs are involved in 3′ splice site selection in mammalian introns. Mol Cell Biol.

[pgen-0020045-b028] Iida Y (1985). Splice-site signals of mRNA precursors as revealed by computer search. Site-specific mutagenesis and thalassemia. J Biochem.

[pgen-0020045-b029] Carninci P, Kasukawa T, Katayama S, Gough J, Frith M (2005). The transcriptional landscape of the mammalian genome. Science.

[pgen-0020045-b030] van Nimwegen E, Paul N, Sheridan R, Zavolan M (2006). SPA: A probabilistic algorithm for spliced alignment. PLoS Genet.

