# Supplementary material for: A Simple Physical Model Predicts Small Exon Length Variations
Source: PLoS Genet. 2006 Apr 28;2(4):e45. doi: 10.1371/journal.pgen.0020045 (PMC1449888; doi:10.1371/journal.pgen.0020045)
Supplement: Table S1 — Note that, in order to correct for NMD, the number of variations of length three has been multiplied by 0.53 and rounded to the nearest integer. (11 KB PDF) [file pgen.0020045.st001.pdf]

Supporting Table S1: Frequences of small exon-length variations

Tzu-Ming Chern   Erik van Nimwegen   Chikatoshi Kai   Jun Kawai
Piero Carninci   Yoshihide Hayashizaki   Mihaela Zavolan

December 10, 2005

splice site	perc. 1 (num.)	perc. 2 (num.)	perc. 3 (num.)	perc. 4 (num.)
CDS acceptor	0.19 ± 0.032	0.08 ± 0.022	0.6 ± 0.04	0.13 ± 0.027
UTR acceptor	0.13 ± 0.063	0.09 ± 0.053	0.5 ± 0.093	0.29 ± 0.085
NC acceptor	0.13 ± 0.14	0.07 ± 0.11	0.6 ± 0.20	0.2 ± 0.167
CDS donor	0.32 ± 0.077	0.24 ± 0.071	0.12 ± 0.071	0.32 ± 0.077
UTR donor	0.29 ± 0.13	0.14 ± 0.097	0.05 ± 0.061	0.51 ± 0.14
NC donor	0.38 ± 0.34	0.38 ± 0.34	0 ± 0	0.25 ± 0.31XS

The relative frequencies and two standard errors of exon-length variations of length 1 through 4 at donor and acceptor sites of different exons types. Note that, in order to correct for NMD, the number of variations of lengths 3 has been multiplied by 0.53 and rounded to the nearest integer.
